# Psychological Morbidity among University Students in Hong Kong (2014–2018): Psychometric Properties of the Depression Anxiety Stress Scales (DASS) and Related Correlates

**DOI:** 10.3390/ijerph18168305

**Published:** 2021-08-05

**Authors:** Xiang Li, Daniel T. L. Shek, Esther Y. W. Shek

**Affiliations:** Department of Applied Social Sciences, The Hong Kong Polytechnic University, Hong Kong, China; daniel.shek@polyu.edu.hk (D.T.L.S.); esther.shek@polyu.edu.hk (E.Y.W.S.)

**Keywords:** depression, anxiety, stress, positive youth development, life satisfaction, university students

## Abstract

Although mental health problems among Hong Kong university students are serious, there is a lack of studies examining the psychometric properties of related assessment scales and correlates. This study attempted to validate the Depression Anxiety Stress Scales (DASS) in Hong Kong university students and examine the demographic (gender), time (cohort), and well-being correlates (positive youth development attributes and life satisfaction) of psychological morbidity. Confirmatory factor analysis (CFA) was used to examine the factor structure of the DASS (*n* = 6704). Gender and cohort invariance were further established using a multigroup CFA. The three-factor model of the DASS showed a superior fit and factorial invariance across gender and five different cohorts. Regarding gender and cohort correlates of psychological morbidity, males exhibited more depression, anxiety, and stress symptoms than their female counterparts. The intensity of psychological distress also escalated after the Umbrella Movement in 2014. Furthermore, well-being measures (positive youth development and life satisfaction) were negatively associated with depression, anxiety, and stress. In short, the Chinese DASS demonstrated good psychometric properties. This study also showed that gender, cohort (occurrence of political events), and well-being were associated with psychological morbidity indexed by the DASS measures.

## 1. Introduction

Adolescent mental health problems are a growing concern [1]. Depression, anxiety, and stress are important indicators of common mental health problems [2,3]. In past decades, an upward trend of psychological distress has been reported, with an increasing number of young people suffering from mental health problems all around the world [4,5,6,7]. In a meta-analysis of 24 studies, Regehr [8] reported that around half of university students displayed moderate levels of mental health problems such as depression, anxiety, and stress. In the United States [5], researchers found that 33% of university students exhibited depressive symptoms, 40% exhibited anxiety symptoms, and 38% showed symptoms of stress. In mainland China, Gao [6] found that 32%, 45%, and 26% of university students exhibited depression, anxiety, and stress, respectively. The high prevalence of psychological morbidity among university students is alarming and deserves further investigation and intervention.

Hong Kong is a highly competitive city where young people face huge stress such as academic/career challenges, high property prices, and low income [1], with a significant number of young locals displaying psychological distress [9]. A 10-year longitudinal cohort study indicated that the percentage of the Hong Kong adult population with depression increased from 1.9% between 2009 and 2014 to 11.2% in 2019 [10]. Moreover, many studies have found that mental health problems and psychological morbidity are quite common among university students [5,7,8,9,11]. In a study of 7915 freshmen in Hong Kong, Wong et al. [9] found that 21%, 41%, and 27% of students displayed moderately severe or above-average levels of depression, anxiety, and stress levels, respectively. Similarly, in a survey involving 1200 university students in Hong Kong, 68.5% of participants reported mild to severe depression symptoms, and 54.4% reported mild to severe symptoms of anxiety [11]. Due to the high lifetime prevalence of psychological distress and the great suffering of patients and society, it is urgent to have a deeper understanding of university students’ mental health.

There are two demographic correlates of the psychological morbidity of university students which deserve attention. First, gender differences in psychological morbidity have long been a focus of researchers, but the findings are mixed. Although many traditional viewpoints have supposed that females are more vulnerable to negative events and have more mental health problems than males in general [12,13], an increasing number of studies have argued that males suffer from more burnout and psychological distress than females in the contemporary world (e.g., [14,15]). Specifically, although some scholars found that females are more depressed than males (e.g., [16,17,18,19]), a comparable number of research findings discovered that males reported higher scores on depression scales than females (e.g., [6,9,15,19,20]). In regard to anxiety, most of the existing studies have revealed a higher level of anxiety in females (e.g., [4,6,9,17,21]), while very few studies have found that males were more anxious than females in response to controllable events (e.g., [12]). Concerning stress, some studies have reported that females showed a higher level of stress than males (e.g., [4,9,18,21]), whereas a limited number of studies have indicated that males are more stressed than females (e.g., [15]). In short, the gender difference in mental health is unclear.

Secondly, the occurrence of political events is another correlate of the mental health of young people. Obviously, the occurrence of political events, such as protests, riots, revolutions, or political instability, would also constitute risk factors for adolescent development [22,23]. Thus, different cohorts of young people may display different levels of psychological morbidity in response to the political events they experience. Unfortunately, few studies have examined how political factors may influence adolescent development [24].

Besides gender and political event correlates, there are well-being correlates of psychological morbidity. Conceptually speaking, there are two domains of well-being [25], including eudaimonic well-being (i.e., self-realization and fully functioning) and hedonic well-being (i.e., pleasure-seeking and avoidance of pain). Although some research has shown a relationship between negative events and mental health problems, very few studies have investigated psychological morbidity and well-being indexed by positive youth development (PYD) attributes and life satisfaction. PYD can be regarded as eudaimonic well-being [26], which refers to individuals’ psychosocial competence and positive functioning, which can protect against negative emotions and behaviors [27,28]. Lerner et al. [29] stated that young people with positive development are less likely to experience mental health problems such as depression. Zhou et al. [30] uncovered that PYD attributes could protect young people from depression. Although the benefits of PYD have been widely studied among adolescents, they have seldom been explored in university students [28,31].

Life satisfaction can be regarded as hedonic well-being [26,32], and the impact of life satisfaction on depression, anxiety, and stress has been reported (e.g., [33,34]). For instance, Gilman and Huebner [35] and Tsitsas et al. [36] revealed that life satisfaction had negative impacts on depression, anxiety, and stress. However, most of the existing studies are based on Western people [35,36].

In view of the growing mental health problems in university students, the development of objective assessment tools is indispensable. In the scientific literature, some scales have been commonly used to measure mental health problems, but only the DASS can measure depression, anxiety, and stress using a single instrument. The DASS has been translated into at least 44 different languages and has been validated across different age groups, different countries, different regions, and clinical and non-clinical populations [37]. Although some researchers have argued that either the one-factor model [19], the bi-factor model [17], or the second-order model/hierarchical factor model [38] is more suitable than the original three-factor model [39], most studies have supported the three-factor model (e.g., [40,41,42,43]).

Although the DASS has been validated in mainland China [20,42], it has not been validated in other Chinese communities, including Singapore, Taiwan, Macau, and Hong Kong. Among the existing Chinese studies using the DASS, Wong et al. [9] revealed that male university students displayed more depression and less anxiety and stress than their female counterparts in Hong Kong. Gao et al. [6] found that female mainland Chinese university students were significantly more anxious than males, with no gender differences in terms of depression and stress. In short, Chinese findings concerning gender differences in mental health are equivocal. Furthermore, very limited research has examined the role of political events in adolescent psychological morbidity. Due to the de-emphasis of holistic development in Chinese communities [1], only very limited Chinese studies investigated the role of PYD and life satisfaction in reducing psychological distress, although PYD has been demonstrated to positively prevent psychological and social problems [30,44]. Finally, no study has to date examined the relationship between well-being (indexed by PYD and life satisfaction) and psychological morbidity (indexed by the DASS).

To fill these research gaps, this study examined the factor structure of the DASS in Hong Kong and also examined the gender, political event, and well-being (PYD and life satisfaction) correlates and psychological morbidity indexed by the DASS measures. We attempted to address the following research questions.

Research Question 1: What is the mental health status of university students in Hong Kong? With reference to the findings that mental health symptoms are widespread in university students in Hong Kong [7,9,10,11], we expected that prevalence rates based on the DASS would be comparable to the existing findings.

Research Question 2: What are the psychometric properties of the DASS in university students in Hong Kong?

With reference to the literature, we put forward the following two hypotheses:

**Hypothesis** **1.** *It was expected that three factors (depression, anxiety, and stress) would emerge from the data based on the DASS*.

**Hypothesis** **2.** *It was expected that the DASS would show stable factor structure across gender and time (five cohorts from 2014/15 to 2018/19 academic years) (Hypothesis 2a and Hypothesis 2b, respectively)*.

Research Question 3: Is gender related to the psychological morbidity of university students in Hong Kong? Based on the existing research findings [9], it was expected that male students would show more symptoms of depression, anxiety, and stress than their female counterparts (Hypothesis 3).

Research Question 4: Do different cohorts of students show different levels of psychological morbidity?

As the political situation in Hong Kong became stressful after 2014 (i.e., Umbrella Movement), we expected that the depression, anxiety, and stress symptoms would increase in the cohorts in 2015/16 to 2018/19 academic year (Hypothesis 4).

Research Question 5: Is psychological morbidity related to different aspects of well-being?

Based on the literature [44,45,46], it was expected that a higher level of eudaimonic well-being (indexed by PYD) and a higher level of hedonic well-being (indexed by life satisfaction) would be negatively related to depression, anxiety, and stress (Hypothesis 5a and Hypothesis 5b, respectively).

## 2. Materials and Methods

### 2.1. Participants

We collected data from students at the Hong Kong Polytechnic University. These students took a General University Requirements course entitled “Tomorrow’s Leaders” in the 2014/15, 2015/16, 2016/17, 2017/18, and 2018/19 academic years. Except for students in the Faculty of Business, all Year 1 students at this university are required to take this subject.

### 2.2. Procedures

We obtained ethical approval from the university’s institutional review board. Participation was voluntary, and there was no penalty for students if they refused to participate in this study. Written informed consent was obtained from each participant prior to data collection.

### 2.3. Measures

#### 2.3.1. Depression Anxiety Stress Scales [DASS]

The 21-item DASS [39] includes three subscales: Depression (e.g., “I could not seem to experience any positive feelings at all”), Anxiety (e.g., “I felt scared without good reason”), and Stress (e.g., “I found it difficult to relax”). Items are rated on a four-point scale from 0 (*not at all*) to 3 (*most of the time*), with higher scores suggesting more mental health problems. Lovibond and Lovibond [39] classified the severity of depression, anxiety, and stress into five categories including normal, mild, moderate, severe, and extremely severe. This scale has demonstrated excellent internal consistency in this study: 0.90, 0.86, and 0.88 for depression, anxiety, and stress, respectively. Both English and Chinese versions of the DASS have been used in Hong Kong [9]. As English is the teaching medium at the Hong Kong Polytechnic University, the English version of the DASS was used in this study.

#### 2.3.2. The Chinese Positive Youth Development Scale [CPYDS]

The CPYDS [47] was used to measure the PYD construct of the participants. Specifically, 34 core items around the PYD attributes (Catalano et al., 2004) were rated on a six-point scale from 1 (*strongly disagree*) to 6 (*strongly agree*), with higher scores indicating greater PYD. This scale has demonstrated excellent internal consistency (*a* = 0.95) in this study.

#### 2.3.3. The Satisfaction with Life Scale [SWLS]

The SWLS [48] was used to measure students’ perceptions of their quality of life. It covers five items (e.g., “I am satisfied with my life”). Students rated each item from 1 (*strongly disagree*) to 6 (*strongly agree*), with a higher score suggesting a higher level of life satisfaction. The present sample’s Cronbach alpha for life satisfaction was very good (*a* = 0.87). This scale has been used in Hong Kong [49].

### 2.4. Data Analysis Plan

To examine the factor structure of the DASS, confirmatory factor analysis was performed using Mplus 8 [50]. As the DASS items are ordered categorically, weighted least squares (WLS) estimation was employed, due to its good fit for ordinal data [51]. Four models were tested: the null model, the one-factor model, the three-factor model, and the secondary-factor model (three primary factors of depression, anxiety, and stress and one secondary factor of psychological distress). Notably, the bi-factor model was not included in our test list due to its limitations such as its tendency to overfit and problems concerning the interpretation of orthogonal latent factors [20,52]. Each item was constrained to load only on one factor. A combination of fit indices, including the comparative fit index (CFI), the Tucker-Lewis index (TLI), and the root mean square error of approximation (RMSEA), were used to evaluate the model fit. CFI ≥ 0.95, TLI ≥ 0.95, and RMSEA ≤ 0.08 are considered a good fit [53,54].

For factor invariance, the best-fitting baseline model was first tested separately for males and females [55]. In the baseline model (i.e., configural invariance), no equality constraints were imposed. Following that, a series of progressively restrictive invariance models constrained factor loadings (weak invariance), item intercepts (strong invariance), error variances (strict invariance), and factor variances (structure invariance) equally across gender [56]. For gender differences on the DASS scores, we tested latent factor means in the male and female participants. We conducted the same procedures to test the measurement and structural invariance of the three-factor DASS model across the five academic years, and the year differences across the latent factor mean. A chi-square difference test (i.e., Δχ^2^) is typically employed to measure the degree of invariance between unconstrained and constrained models, but this test is oversensitive to sample size and is over-stringent. Instead, ΔCFI ≤ 0.01 [57] and ΔRMSEA ≤ 0.015 [58] can function as the criteria indicating factorial invariance. The influence of PYD and life satisfaction on the DASS scores were tested by structural equation modeling (SEM).

## 3. Results

Between the 2014/15 and 2018/19 academic years, 9832 first-year students at the Hong Kong Polytechnic University took this subject and 68.2% of them (*n* = 6704) participated in this study. Specifically, in the 2014/15 academic year, 1669 out of 2083 students (80.1%) joined this study; in the 2015/16 academic year, 1584 out of 2127 students (74.5%) joined this study; in the 2016/17 academic year, 1568 out of 2112 students (74.2%) joined this study; in the 2017/18 academic year, 849 out of 1650 students (51.5%) joined this study; and in the 2018/19 academic year, 1034 out of 1860 students (55.6%) joined this study. Of all the participants, 45.2% were male (*n* = 3031), 54.5% were female (*n* = 3655), and 0.3% (*n* = 18) did not disclose their gender. In terms of age, 93.3% of the respondents were 18 years or older (*n* = 6252), and 56.2% of the students were 18 years old (*n* = 3769). Details are reported in Table 1.

### 3.1. Psychological Morbidity in Hong Kong University Students

The mean values, standardized deviations, reliabilities, and correlations among all the study variables are presented in Table 2. Depression, anxiety, and stress are closely related to each other: depression and anxiety, r = 0.77 (*p* < 0.001); depression and stress, r = 0.80 (*p* < 0.001); and anxiety and stress, r = 0.84 (*p* < 0.001). The mean values for the depression, anxiety, and stress scales were 0.72, 0.78, and 0.90, respectively, indicating a mild depression level, a moderate anxiety level, and a normal stress level according to the criteria set by Lovibond and Lovibond [39] and adopted in Hong Kong [9,59]. Additionally, the severity distribution (see Table 3) revealed that depression (47.6%, 95% CI 46.4–48.9), anxiety (65%, 95% CI 63.8–66.1), and stress (32.2%, 95% CI 31.1–33.4) were prevalent in the current sample. Moreover, 32.7% (95% CI 31.6–33.9), 44.7% (95% CI 43.5–45.9), and 18.5% (95% CI 17.6–19.5) of students showed moderate or above average levels of depression, anxiety, and stress, respectively.

### 3.2. Confirmatory Factor Analysis of the DASS

The three-factor model and the second-order model showed a superior model fit to the one-factor and null models (see Table 4). The three-factor model and second-order model showed the same model fit; nevertheless, the three-factor model was retained as a more parsimonious model with theoretical support. The overall fit of the three-factor DASS model was excellent: χ^2^ (186, *n* = 6704) = 6638, *p* < 0.001, CFI = 0.964, TLI = 0.960, and RMSEA = 0.072 (90% CI: 0.070–0.073). All factor loadings were high, ranging from 0.71 to 0.88 for depression, 0.55 to 0.87 for anxiety, and 0.72 to 0.85 for stress. Therefore, the three-factor model was preferable. Hence, Hypothesis 1 was supported.

### 3.3. Gender Invariance of the DASS

To test the invariance across gender, the independent best-fitting models for the male and female participants were first established. The three-factor model of the DASS was good for both the male participants, χ^2^ (186, *n* = 3031) = 3052, *p* < 0.001, CFI = 0.972, TLI = 0.968, and RMSEA = 0.071 (90% CI: 0.069–0.074), and the female participants, χ^2^ (186, *n* = 3655) = 3478, *p* < 0.001, CFI = 0.960, IFI = 0.954, and RMSEA = 0.070 (90% CI: 0.068–0.072). Thereafter, five successively restrictive invariance models across gender were tested (see Table 5); the results supported the gender invariance of the three-factor structure (i.e., Hypothesis 2a) with ΔCFI ≤ 0.01 and ΔRMSEA ≤ 0.015.

### 3.4. Cohort Invariance of the DASS

To test the invariance of the three-factor DASS model across the five academic years, the independent best-fitting models in the five cohorts were first established. The three-factor model was shown to be a good fit for students in the 2014/15, 2015/16, 2016/17, 2017/18, and 2018/19 academic years (see Table 6). Likewise, five successively restrictive invariance models across academic years were tested. The results supported the invariance of the three-factor structure across the five cohorts (Hypothesis 2b) with ΔCFI ≤ 0.01 and ΔRMSEA ≤ 0.015.

### 3.5. Gender Differences in Mental Health

Latent mean comparisons were conducted using the male participants as the reference group. All mean differences between the male and female participants were significant (*p* < 0.001); the male participants had significantly higher levels of depression (*M*_Diff_ = −0.40), anxiety (*M*_Diff_ = −0.26), and stress (*M*_Diff_ = −0.19) than female participants. Thus, Hypothesis 3 was supported.

### 3.6. Cohort Differences in Mental Health

**Figure 1:**
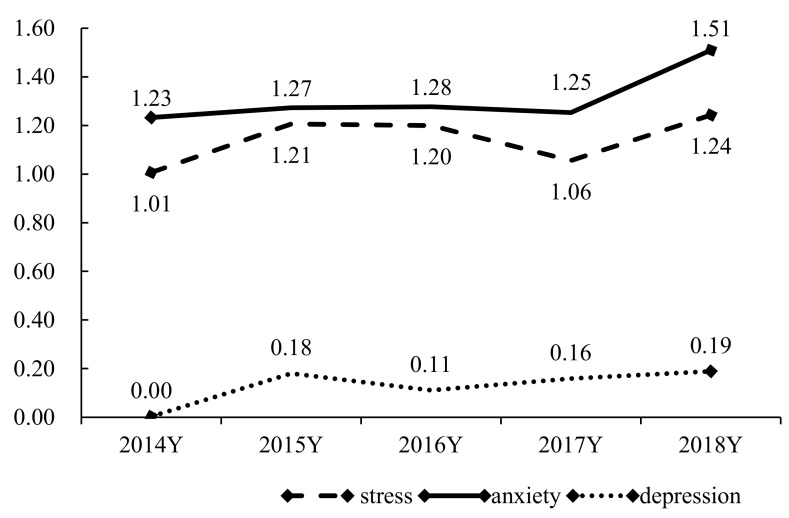
The trends of depression, anxiety, and stress from 2014/15 to 2018/19 academic years. Note. Latent means in each year have been indicated in this figure.

We further tested the latent mean differences across the five cohorts, with the 2014 cohort as the reference group (see Table 7). Students in the 2014 cohort showed significantly lower levels of depression, anxiety, and stress compared to the following four cohorts, while no significant differences in latent mean scores were found among the four cohorts from 2015 to 2018 (see Figure 1). Hence, Hypothesis 4 was confirmed.

### 3.7. The Relationship between Well-Being and Psychological Morbidity

**Figure 2:**
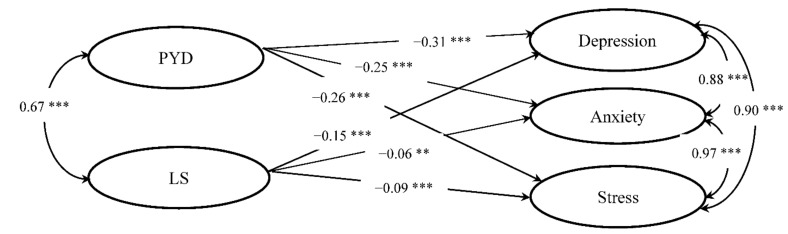
Structural equation model. Note. Latent constructs are shown in ellipses. PYD = positive youth development; LS = life satisfaction. ** *p* < 0.01; *** *p* < 0.001.

The SEM results revealed that the different measures of well-being (i.e., PYD and life satisfaction) were significantly related to the university students’ depression, anxiety, and stress. The structural model (see Figure 2) fit the data very well: χ^2^ (655, *n* = 6704) = 10,404, *p* < 0.001, with CFI of 0.949, TLI of 0.946, and RMSEA of 0.047 (90% CI: 0.046–0.048). Specifically, while PYD showed a significant negative relationship with depression (β = −0.31, *p* < 0.001), anxiety (β = −0.25, *p* < 0.001), and stress (β = −0.26, *p* < 0.001), life satisfaction also showed significant a negative relationship with depression (β = −0.15, *p* < 0.001), anxiety (β = −0.06, *p* < 0.01), and stress (β = −0.09, *p* < 0.001). In short, students with higher levels of PYD and life satisfaction displayed less psychological morbidity, supporting Hypotheses 5a and 5b.

## 4. Discussion

Mental health problems appeared to be more serious in Hong Kong university students than 15 years ago [9]. The mean depression has moved from “normal” to “mild”, the mean anxiety has moved from “mild” to “moderate”, and only the mean of stress stayed within a “normal” level among university students. Meanwhile, more young students showed at least moderate levels of depression (32.7% vs. 21%) and anxiety (44.7% vs. 41%). These prevalence rates suggest that the mental health scores of university students under the current study are worse than those reported previously. This implies that the psychological distress of young university students in Hong Kong deserves notable attention and relevant and adequate mental health services should be provided.

Consistent with the findings of a systematic review covering 45 studies [37], the three-factor DASS model exhibited the best fit and provided a better model fit than other proposed models, hence supporting Hypothesis 1. This implies that among Chinese young people, the construct of psychological morbidity can be well distinguished by three dimensions: depression, anxiety, and stress. The good psychometric properties of the DASS demonstrate that this scale can measure psychological morbidity in an objective manner in a Chinese context.

In line with previous studies (e.g., [60]), both the measurement and structural invariance across gender were established in this study. This study demonstrates the configural, metric, scalar, error variance, and factor variance invariance across gender in a Chinese sample, suggesting that the DASS-21 is an appropriate instrument to measure the same constructs of depression, anxiety, and stress across gender. Hypothesis 2a was supported. Male and female university students conceptualize and interpret the three dimensions of psychological distress in a similar way. The findings suggest that the DASS-21 will not be affected by gender in a non-clinical setting, and gender differences are due to a true difference in the construct of psychological distress rather than different psychometric responses to the scale items [57]. Furthermore, the structural invariance of the DASS across the five different cohorts was also established in this study, suggesting that the factor structure does not change across time. These findings support Hypothesis 2b. Taken as a whole, the present study provides good support for the psychometric properties of the DASS in university students in Hong Kong. It is noteworthy that the analyses used in this study are consistent with those reported in the field [41,42,43,61,62].

Regarding the socio-demographic correlates of the DASS, we found that male university students displayed significantly more depression, anxiety, and stress symptoms than did female university students which provides support for Hypothesis 3. These findings are in line with the existing literature [9]. As social norms discourage males from openly expressing negative internal emotions and psychological distress [63], this may explain the relatively higher psychological morbidity in male students. A meta-analysis with 205 studies [64] indicated that, compared with females, males seem to feel more obliged to hide their inner thoughts and experiences and therefore tend to do so. The low level of emotional disclosure and the lower level of support-seeking among males may also explain why males are more depressed than females.

In contrast to most existing studies (e.g., [6]) but consistent with very few studies (e.g., [65]), male students were found to have a higher level of anxiety than their female counterparts. Males are found to be more anxious than females when it comes to learning and examinations [65]. This may explain why males would feel more anxious than females in a university context where learning and taking examinations are the most important and major duties for students.

Finally, we also found that the male university students exhibited a higher level of stress than their female counterparts. In Hong Kong, there are more males (67.5%) than females (55%) in the labor market [66] because males are expected to take more responsibility for earning money and supporting their families in Chinese society [67]. Thus, male university students may worry more about their future careers after graduation and suffer more stress than their female peers. Although males have shown significantly more depression, anxiety, and stress than females, evidence has shown that compared with females, males tend to solve their emotional problems themselves and to use fewer mental health services [6,7,68,69]. Therefore, we should pay more attention to promoting the mental health of male university students.

Consistent with Hypothesis 4, we found that psychological morbidity escalated after 2014 (i.e., occurrence of a political event). Although we found no significant changes in psychological morbidity between the 2015/16 and 2018/19 academic years, the depression, anxiety, and stress of university students increased year by year, and psychological morbidity reported by students in 2018 was significantly higher than that reported in 2014. This trend of increasing levels of psychological morbidity among university students may reflect the social instability in Hong Kong during the past few years. An important turning point in Hong Kong’s social life was the civil disobedience campaign “Umbrella Movement”, which took place in 2014. In an investigation based on a population-representative sample of 1,208 Hong Kong people in 2015 [16], Hou et al. revealed that in 2015, Hong Kong citizens experienced significantly higher levels of depression and anxiety than they did in 2003 [70] and 2011 [71]. Ni et al. [72] also found that major depression increased by 7% in Hong Kong adults after the Umbrella Movement. This suggests that young people in Hong Kong have been suffering more and more psychological distress since 2014. Existing studies (e.g., [18,73]) found that stressful life events, such as social movements, make people depressed, and politics may have become a regular and persistent risk factor that negatively affects mental health [74]. Therefore, timely intervention on the psychological morbidity of university students in connection with political events in Hong Kong is required. In view of the paucity of research on the experience of political events and psychological morbidity in university students in Hong Kong, this study is a valuable contribution to the limited literature.

As expected, PYD as an indicator of eudaimonic well-being, Martela and Sheldon [32] showed significant and negative impacts on the three factors of psychological distress in our samples. This finding can be supported by existing studies that have demonstrated that good PYD can relieve individuals’ psychological distress (e.g., [44,45,75,76]). PYD is able to nurture youth mental health and encourage young people to make positive changes in multiple domains of their life to prevent behavioral and emotional problems [77]. Consistent with previous studies (e.g., [46,78]), we also found that life satisfaction as an indicator of hedonic well-being has significant negative impacts on depression, anxiety, and stress. As people with higher levels of life satisfaction may have positive values and are more resistant to mental health disorders, life satisfaction is an important factor in protecting individuals’ mental health [33]. Life satisfaction can help people to maintain positive mental functioning by enhancing internal motivation in stressful situations, while a low level of life satisfaction is a major risk factor for mental health problems, such as depression [79]. In short, the findings provide support for Hypotheses 5a and 5b that both eudaimonic and hedonic well-being are important correlates of psychological morbidity in young people. Promoting PYD and increasing life satisfaction deserve the attention of researchers and should be taken into account when combating mental health problems in young people.

Five limitations should be acknowledged and addressed in future work. First, although the sample size in the present study was large, all respondents were from the same university. The findings cannot be generalized to all university students in Hong Kong, and need to be replicated in other universities in Hong Kong to avoid sampling bias. Moreover, one should be careful not to generalize the findings in this study to Western society, due to potential significant language effects when measuring psychological distress [80]. Second, multiple variables could be related to mental health problems, such as psychological factors, environmental factors, temperamental characteristics, and coping strategies [81]. However, we only tested gender, political event, and well-being correlates with mental health problems in this study. The association between different factors and mental health problems should be examined in the future. Third, we did not have data for other age groups (e.g., children and elders) and clinical samples. In the future, furthe exploration of psychological morbidity across different age groups and different populations is necessary. Fourth, although we recruited five cohorts of university students in this study, this study is essentially a cross-sectional investigation, which only allows us to understand associations, not causality, between well-being correlates and psychological mobility. A longitudinal study would be helpful to reveal the causal relationships amongst the variables under investigation. Last but not least, we did not test psychological distress in the 2019/20 academic year that could reflect the mental health of young people after the social unrest in Hong Kong. Future work would benefit from examining students’ mental health after the social unrest in 2019 as many young students were involved in this unrest and social dysfunction would influence young people [82]. With the emergence of COVID-19 in 2020, systematic studies on the quality of life of university students are also indispensable [83,84].

## 5. Conclusions

This study makes the screening and diagnosis of young people with psychological morbidity possible by validating the use of the DASS in Hong Kong university students. This study showed that the severity of students depression, anxiety, and stress symptoms deserves attention. In terms of the psychometric properties of the scale, the present findings showed that the DASS is a valid and sound instrument across genders and cohorts which can be used by clinical professionals to identify psychological distress and to design appropriate intervention programs for university students. The findings of this study also extend the cross-cultural understanding of mental health by discovering the association between well-being correlates and psychological morbidity. There is a strong need for professionals to promote PYD and life satisfaction in interventions for young people with psychological morbidity.

## Figures and Tables

**Table 1:** Demographic information of all participants (*n* = 6704).

Demographic Variables	Frequency	Valid Percent
Academic year		
2014–15	1669	24.9
2015–16	1584	23.6
2016–17	1568	23.4
2017–18	849	12.7
2018–19	1034	15.4
Gender		
Male	3031	45.3%
Female	3655	54.7%
Age		
16 or below	12	0.2%
17-year-old	432	6.5%
18-year-old	3769	56.3%
19-year-old	1461	21.8%
20 or above	1022	15.3%
Place of birth		
Hong Kong	4765	71.2%
Mainland China	1429	21.4%
Other places	493	7.4%
School/Faculty		
School of Design	259	3.9%
Faculty of Humanities	348	5.2%
Faculty of Construction and Environment	956	14.3%
Faculty of Engineering	1360	20.3%
Faculty of Hotel and Tourism Management	482	7.2%
Faculty of Health and Social Sciences	2393	35.7%
Faculty of Applied Science and Textiles	904	13.5%

**Table 2:** Means, Standard Deviations, Reliabilities, and Correlations of Study Variables in this Study.

	All	Male	Female	1	2	3	4	5
	Mean (SD)	Mean (SD)	Mean (SD)					
Depression	0.72 (0.62)	0.84 (0.68)	0.62 (0.54)	-				
Anxiety	0.78 (0.57)	0.85 (0.62)	0.72 (0.51)	0.77 ***	-			
Stress	0.90 (0.60)	0.95 (0.65)	0.86 (0.55)	0.80 ***	0.84 ***	-		
PYD	4.64 (0.53)	4.61 (0.58)	4.67 (0.48)	−0.37 ***	−0.25 ***	−0.28 ***	-	
LS	3.91 (0.92)	3.78 (0.98)	4.02 (0.85)	−0.30 ***	−0.18 ***	−0.22 ***	0.59 ***	-

Note. *** *p* < 0.001. PYD = positive youth development; LS = life satisfaction.

**Table 3:** Severity Distribution of Depression, Anxiety, and Stress in this Study.

Variables	Normal	Mild	Moderate	Severe	Extremely Severe
Depression	3510 (52.4%)	999 (14.9%)	1471 (21.9%)	343 (5.1%)	381 (5.7%)
Anxiety	2349 (35%)	1358 (20.3%)	1323 (19.7%)	747 (11.2%)	927 (13.8%)
Stress	4543 (67.7%)	921 (13.7%)	648 (9.7%)	433 (6.5%)	159 (2.4%)

**Table 4:** Confirmatory Factor Analysis (CFA) of the DASS and the Factor Loadings.

Models	χ^2^	df	CFI	TLI	RMSEA (90% CI)		
Null model	181,141	210	0.000	0.000	0.358 (0.357–0.360)		
One-factor	9874	189	0.946	0.941	0.087 (0.086–0.089)		
Three-factor	6638	186	0.964	0.960	0.072 (0.070–0.073)		
Second-order	6638	186	0.964	0.960	0.072 (0.070–0.073)		
					Factor Loadings		
DASS Items					All	Male	Female
Depression							
3. I could not seem to experience any positive feeling at all					0.82	0.83	0.79
5. I found it difficult to work up the initiative to do things					0.71	0.73	0.68
10. I felt that I had nothing to look forward to					0.84	0.85	0.82
13. I felt down-hearted and blue					0.88	0.88	0.88
16. I was unable to become enthusiastic about anything					0.83	0.84	0.81
17. I felt I was not worth much as a person					0.87	0.88	0.84
21. I felt that life was meaningless					0.83	0.85	0.79
Anxiety							
2. I was aware of dryness of my mouth					0.55	0.59	0.49
4. I experienced breathing difficulty					0.75	0.78	0.70
7. I experienced trembling					0.71	0.76	0.63
9. I was worried about situations in which I might panic and make a fool of myself					0.78	0.79	0.79
15. I felt I was close to panic					0.87	0.88	0.86
19. I was aware of the action of my heart in the absent of physical exertion					0.75	0.79	0.69
20. I felt scared without good reason					0.81	0.84	0.78
Stress							
1. I found it hard to wind down.					0.72	0.74	0.70
6. I tend to over-react to situations					0.74	0.78	0.69
8. I felt that I was using lot of nervous energy					0.81	0.83	0.80
11. I found myself getting agitated					0.85	0.87	0.82
12. I found it difficult to relax					0.83	0.83	0.82
14. I was intolerant of anything that kept me from getting on with what I was doing					0.79	0.82	0.76
18. I felt that I was rather touchy					0.74	0.78	0.70

Note. All χ^2^ values are statistically significant (*p* < 0.001). All factor loadings are statistically significant (*p* < 0.001).

**Table 5:** Testing for gender invariance: Results of the three-factor model of the DASS.

	Overall Fit Indices							
Models	χ^2^	CFI		TLI	RMSEA (90% CI)			
Male	3052	0.972		0.968	0.071 (0.069–0.074)			
Female	3478	0.960		0.954	0.070 (0.068–0.072)			
	Overall fit indices				Comparative fit indices			
Models	χ^2^	CFI	TLI	RMSEA (90% CI)	Comparison	Δχ^2^	ΔCFI	ΔRMSEA
Model 1	6544	0.966	0.962	0.070 (0.069–0.072)				
Model 2	6406	0.967	0.965	0.068 (0.066–0.069)	M2 VS. M1	134 ***	0.001	0.002
Model 3	6472	0.967	0.968	0.065 (0.064–0.066)	M3 VS. M2	405 ***	0.000	0.003
Model 4	4821	0.976	0.978	0.054 (0.053–0.055)	M4 VS. M3	127 ***	0.009	0.011
Model 5	4230	0.979	0.981	0.050 (0.049–0.051)	M5 VS. M4	123 ***	0.003	0.004

Note. Model 1: configural invariance; Model 2: metric invariance; Model 3: scalar invariance. Model 4: error variance invariance; Model 5: factor variance invariance. *** *p* < 0.001. All χ^2^ are WLSMV χ^2^. WLSMV χ^2^ difference tests were conducted via the Mplus’ DIFFTEST function (Asparouhov and Muthén, 2006).

**Table 6:** Testing for samples of cohort invariance: Results of the three-factor model of the DASS.

	Overall Fit Indices							
Models	χ^2^	CFI		TLI	RMSEA (90% CI)			
2014	1833	0.967		0.963	0.073 (0.070–0.076)			
2015	1922	0.962		0.957	0.077 (0.074–0.080)			
2016	1542	0.969		0.964	0.068 (0.065–0.071)			
2017	919	0.969		0.966	0.068 (0.064–0.073)			
2018	1045	0.962		0.957	0.067 (0.063–0.071)			
	Overall fit indices				Comparative fit indices			
Models	χ^2^	CFI	TLI	RMSEA (90% CI)	Comparison	Δχ^2^	ΔCFI	ΔRMSEA
Model 1	7057	0.968	0.963	0.070 (0.069–0.072)				
Model 2	6612	0.970	0.969	0.065 (0.063–0.066)	M2 VS. M1	73	0.002	0.005
Model 3	6303	0.973	0.975	0.058 (0.056–0.059)	M3 VS. M2	190 *	0.003	0.007
Model 4	4798	0.981	0.984	0.046 (0.045–0.048)	M4 VS. M3	177 ***	0.008	0.012
Model 5	3518	0.988	0.990	0.037 (0.035–0.038)	M5 VS. M4	31 **	0.007	0.009

*Note.* Model 1: configural invariance; Model 2: metric invariance; Model 3: scalar invariance. Model 4: error variance invariance; Model 5: factor variance invariance. * *p* < 0.05; ** *p* < 0.01; *** *p* < 0.001. All χ^2^ are WLSMV χ^2^. WLSMV χ^2^ difference tests were conducted via the Mplus’ DIFFTEST function (Asparouhov and Muthén, 2006).

**Table 7:** Latent means comparison for 2015–2018 compared with the 2014 cohort.

Domain	*M*_Diff_	*SE*
Depression ^2015^	0.111 **	0.040
Anxiety ^2015^	0.042	0.040
Stress ^2015^	0.067	0.039
Depression ^2016^	0.105 **	0.040
Anxiety ^2016^	0.046	0.040
Stress ^2016^	0.084 *	0.039
Depression ^2017^	0.138 **	0.047
Anxiety ^2017^	0.059	0.047
Stress ^2017^	0.110 *	0.046
Depression ^2018^	0.132 **	0.044
Anxiety ^2018^	0.087 *	0.044
Stress ^2018^	0.126 **	0.043

Note. * *p* < 0.05, ** *p* < 0.01. SE = standard error; 2014 = reference class with mean = 0 for all domains.

## Data Availability

The data presented in this study are available on request from the corresponding author.

## References

[1] Shek D.T.L., Siu A.M. (2019). “UNHAPPY” environment for adolescent development in Hong Kong. J. Adolesc. Health.

[2] Henry J.D., Crawford J.R. (2005). The short-form version of the depression anxiety stress scales (DASS–21): Construct validity and normative data in a large non-clinical sample. Br. J. Clin. Psychol..

[3] Vignola R.C.B., Tucci A.M. (2014). Adaptation and validation of the depression, anxiety and stress scale (DASS) to Brazilian Portuguese. J. Affect. Disord..

[4] Bayram N., Bilgel N. (2008). The prevalence and socio-demographic correlations of depression, anxiety and stress among a group of university students. Soc. Psychiatry Psychiatr. Epidemiol..

[5] Beiter R., Nash R., McCrady M., Rhoades D., Linscomb M., Clarahan M., Sammut S. (2015). The prevalence and correlates of depression, anxiety, and stress in a sample of college students. J. Affect. Disord..

[6] Gao W., Ping S., Liu X. (2020). Gender differences in depression, anxiety, and stress among college students: A longitudinal study from China. J. Affect. Disord..

[7] Lo S.M., Wong H.C., Lam C.Y., Shek D.T.L. (2020). Common mental health challenges in a university context in Hong Kong: A study based on a review of medical records. Appl. Res. Qual. Life.

[8] Regehr C., Glancy D., Pitts A. (2013). Interventions to reduce stress in university students: A review and meta-analysis. J. Affect. Disord..

[9] Wong J.G., Cheung E.P., Chan K.K., Ma K.K., Tang S.W. (2006). Web-based survey of depression, anxiety and stress in first-year tertiary education students in Hong Kong. Aust. N. Z. J. Psychiatry.

[10] Ni M.Y., Yao X.I., Leung K.S., Yau C., Leung C.M., Lun P., Flores F.P., Chang W.C., Cowling B.J., Leung G.M. (2020). Depression and post-traumatic stress during major social unrest in Hong Kong: A 10-year prospective cohort study. Lancet.

[11] Lun K.W., Chan C.K., Ip P.K., Ma S.Y., Tsai W.W., Wong C.S., Wong T.W., Yan D. (2018). Depression and anxiety among university students in Hong Kong. Hong Kong Med. J..

[12] Thoits P.A. (1987). Gender and marital status differences in control and distress: Common stress versus unique stress explanations. J. Health Soc. Behav..

[13] Van Droogenbroeck F., Spruyt B., Keppens G. (2018). Gender differences in mental health problems among adolescents and the role of social support: Results from the Belgian health interview surveys 2008 and 2013. BMC Psychiatry.

[14] Burke R.J., Greenglass E.R., Schwarzer R. (1996). Predicting teacher burnout over time: Effects of work stress, social support, and self-doubts on burnout and its consequences. Anxiety Stress Coping.

[15] Das P.P.P., Sahoo R. (2012). Stress and depression among post-graduate students. Int. J. Sci. Res..

[16] Hou W.K., Hall B.J., Canetti D., Lau K.M., Ng S.M., Hobfoll S.E. (2015). Threat to democracy: Physical and mental health impact of democracy movement in Hong Kong. J. Affect. Disord..

[17] Jovanović V., Gavrilov-Jerković V., Lazić M. (2019). Can adolescents differentiate between depression, anxiety and stress? Testing competing models of the depression anxiety stress scales (DASS-21). Curr. Psychol..

[18] Liu Y., Zhang N., Bao G., Huang Y., Ji B., Wu Y., Liu C., Li G. (2019). Predictors of depressive symptoms in college students: A systematic review and meta-analysis of cohort studies. J. Affect. Disord..

[19] Jiang L.C., Yan Y.J., Jin Z.S., Hu M.L., Wang L., Song Y., Li N.N., Su J., Wu D.X., Xiao T. (2020). the depression anxiety stress scale-21 in Chinese hospital workers: Reliability, latent structure, and measurement invariance across genders. Front. Psychol..

[20] Wang K., Shi H.S., Geng F.L., Zou L.Q., Tan S.P., Wang Y., Neumann D.L., Shum D.H.K., Chan R.C. (2016). Cross-cultural validation of the Depression Anxiety Stress Scale–21 in China. Psychol. Assess..

[21] Saleh D., Camart N., Romo L. (2017). Predictors of stress in college students. Front. Psychol..

[22] Al-Krenawi A., Graham J.R. (2012). The impact of political violence on psychosocial functioning of individuals and families: The case of Palestinian adolescents. Child Adolesc. Ment. Health.

[23] Garbarino J., Kostelny K. (1996). The effects of political violence on Palestinian children’s behavior problems: A risk accumulation model. Child Dev..

[24] Lin L., Shek D.T.L. (2021). Meaning in life profiles among Chinese late adolescents: Associations with readiness for political participation. Int. J. Environ. Res. Public Health.

[25] Ryan R.M., Deci E.L. (2001). On happiness and human potentials: A review of research on hedonic and eudaimonic well-being. Annu. Rev. Psychol..

[26] Zhou Z., Shek D.T.L., Zhu X. (2020). The importance of positive youth development attributes to life satisfaction and hopelessness in mainland Chinese adolescents. Front. Psychol..

[27] Durlak J.A., Weissberg R.P., Dymnicki A.B., Taylor R.D., Schellinger K.B. (2011). The impact of enhancing students’ social and emotional learning: A meta-analysis of school-based universal interventions. Child Dev..

[28] Dvorsky M.R., Kofler M.J., Burns G.L., Luebbe A.M., Garner A.A., Jarrett M.A., Soto E.F., Becker S.P. (2019). Factor structure and criterion validity of the five Cs model of positive youth development in a multi-university sample of college students. J. Youth Adolesc..

[29] Lerner R.M., Bowers E.P., Geldhof G.J., Gestsdóttir S., DeSouza L. (2012). Promoting positive youth development in the face of contextual changes and challenges: The roles of individual strengths and ecological assets. New Dir. Youth Dev..

[30] Zhou Z., Shek D.T., Zhu X., Dou D. (2020). Positive youth development and adolescent depression: A longitudinal study based on mainland Chinese high school students. Int. J. Environ. Res. Public Health.

[31] Li X., Shek D.T.L. (2020). Objective outcome evaluation of a leadership course utilising the positive youth development approach in Hong Kong. Assess. Eval. High. Educ..

[32] Martela F., Sheldon K.M. (2019). Clarifying the concept of well-being: Psychological need satisfaction as the common core connecting eudaimonic and subjective well-being. Rev. Gen. Psychol..

[33] Brailovskaia J., Schönfeld P., Kochetkov Y., Margraf J. (2019). What does migration mean to us? USA and Russia: Relationship between migration, resilience, social support, happiness, life satisfaction, depression, anxiety and stress. Curr. Psychol..

[34] Tonsing K.N. (2014). Psychometric properties and validation of Nepali version of the Depression Anxiety Stress Scales (DASS-21). Asian J. Psychiatr..

[35] Gilman R., Huebner E.S. (2006). Characteristics of adolescents who report very high life satisfaction. J. Youth Adolesc..

[36] Tsitsas G., Nanopoulos P., Paschali A. (2019). Life satisfaction, and anxiety levels among university students. Creat. Educ..

[37] Lee J., Lee E.H., Moon S.H. (2019). Systematic review of the measurement properties of the Depression Anxiety Stress Scales–21 by applying updated COSMIN methodology. Qual. Life Res..

[38] Ruiz F.J., Martín M.B.G., Falcón J.C.S., González P.O. (2017). The hierarchical factor structure of the Spanish version of Depression Anxiety and Stress Scale-21. Rev. Int. Psicol. Ther. Psicol..

[39] Lovibond P.F., Lovibond S.H. (1995). The structure of negative emotional states: Comparison of the Depression Anxiety Stress Scales (DASS) with the Beck Depression and Anxiety Inventories. Behav. Res. Ther..

[40] Brown T.A., Chorpita B.F., Korotitsch W., Barlow D.H. (1997). Psychometric properties of the Depression Anxiety Stress Scales (DASS) in clinical samples. Behav. Res. Ther..

[41] Crawford J.R., Henry J.D. (2003). The Depression Anxiety Stress Scales (DASS): Normative data and latent structure in a large non-clinical sample. Br. J. Clin. Psychol..

[42] Lu S., Hu S., Guan Y., Xiao J., Cai D., Gao Z., Sang Z., Wei J., Zhang X., Margraf J. (2018). Measurement invariance of the Depression Anxiety Stress Scales-21 across gender in a sample of Chinese university students. Front. Psychol..

[43] Mellor D., Vinet E.V., Xu X., Mamat N.H.B., Richardson B., Román F. (2015). Factorial invariance of the DASS-21 among adolescents in four countries. Eur. J. Psychol. Assess..

[44] Zhu X., Shek D.T.L. (2020). Impact of a positive youth development program on junior high school students in mainland China: A pioneer study. Child. Youth Serv. Rev..

[45] Sarizadeh M.S., Najafi M., Rezaei A.M. (2020). The prediction of depression based on religious coping and the components of positive youth development in adolescents. Ment. Health Relig. Cult..

[46] Fergusson D.M., McLeod G.F.H., Horwood L.J., Swain N.R., Chapple S., Poulton R. (2015). Life satisfaction and mental health problems (18 to 35 years). Psychol. Med..

[47] Shek D.T.L., Siu A.M., Lee T.Y. (2007). The Chinese positive youth development scale: A validation study. Res. Soc. Work Pract..

[48] Diener E.D., Emmons R.A., Larsen R.J., Griffin S. (1985). The satisfaction with life scale. J. Pers. Assess..

[49] Shek D.T.L., Li X. (2016). Perceived school performance, life satisfaction, and hopelessness: A 4-year longitudinal study of adolescents in Hong Kong. Soci. Indic. Res..

[50] Muthén L.K., Muthen B. (2017). Mplus User’s Guide: Statistical Analysis with Latent Variables.

[51] Muthén B. (1984). A general structural equation model with dichotomous, ordered categorical, and continuous latent variable indicators. Psychometrika.

[52] Bornovalova M.A., Choate A.M., Fatimah H., Petersen K.J., Wiernik B.M. (2020). Appropriate use of bifactor analysis in psychopathology research: Appreciating benefits and limitations. Biol. Psychiatry.

[53] Browne M.W., Cudeck R., Bollen K.A., Long J.S. (1993). Alternative Ways of Assessing Model Fit. Testing Structural Equation Models.

[54] Kline R.B. (2015). Principles and Practice of Structural Equation Modeling.

[55] Byrne B.M. (2006). Structural Equation Modeling with EQS: Basic Concepts, Applications and Programming.

[56] Vandenberg R.J., Lance C.E. (2000). A review and synthesis of the measurement invariance literature: Suggestions, practices, and recommendations for organizational research. Oragn. Res. Methods.

[57] Cheung G.W., Rensvold R.B. (2002). Evaluating goodness-of-fit indexes for testing measurement invariance. Struct. Equ. Model..

[58] Chen F.F. (2007). Sensitivity of goodness of fit indexes to lack of measurement invariance. Struct. Equ. Modeling..

[59] Cheung T., Yip P.S. (2016). Lifestyle and depression among Hong Kong nurses. Int. J. Enviorn. Res. Public Health.

[60] Gomez R., Summers M., Summers A., Wolf A., Summers J. (2014). Depression anxiety stress scales-21: Measurement and structural invariance across ratings of men and women. Assessment.

[61] Bravo D.M., Suárez-Falcón J.C., Bianchi J.M., Segura-Vargas M.A., Ruiz F.J. (2021). Psychometric properties and measurement invariance of the maslach burnout inventory–general survey in Colombia. Int. J. Environ. Res. Public Health.

[62] Trigueros R., Navarro-Gómez N., Aguilar-Parra J.M., Cangas A.J. (2020). Factorial structure and measurement invariance of the acceptance and action questionnaire-stigma (AAQ-S) in Spain. Int. J. Environ. Res. Public Health.

[63] Chaplin T.M. (2015). Gender and emotion expression: A developmental contextual perspective. Emot. Rev..

[64] Dindia K., Allen M. (1992). Sex differences in self-disclosure: A meta-analysis. Psychol. Bull..

[65] Janfaza A., Rezaei Y., Soori A. (2014). The relationship between the male and female language performance and the level of anxiety among Iranian EFL students. J. Adv. Linguist..

[66] Census and Statistics Department (2020). Women and Men in Hong Kong.

[67] Zhang C.Z., Guo Q., Mu X. (2016). How female executives affect firm performance? A multi-approach perspective. Adv. Econ. Bus..

[68] Lam L.C.W., Wong C.S.M., Wang M.J., Chan W.C., Chen E.Y.H., Ng R.M.K., Hung S.F., Cheung E.F.C., Sham P.C., Chiu H.F.K. (2015). Prevalence, psychosocial correlates and service utilization of depressive and anxiety disorders in Hong Kong: The Hong Kong mental morbidity survey (HKMMS). Soc. Psychiatry Psychiatr. Epidemiol..

[69] Pattyn E., Verhaeghe M., Bracke P. (2015). The gender gap in mental health service use. Soc. Pyschiatry Psychiatr. Epidemiol..

[70] Leung G.M., Ho L.M., Chan S.K.K., Ho S.Y., Bacon-Shone J., Choy R.Y., Hedley A.J., Lam T.H., Fielding R. (2005). Longitudinal assessment of community psycho-behavioral responses during and after the 2003 outbreak of severe acute respiratory syndrome in Hong Kong. Clin. Infect. Dis..

[71] Nan H., Lee P.H., Ni M.Y., Chan B.H., Lam T.H. (2013). Effects of depressive symptoms and family satisfaction on health related quality of life: The Hong Kong FAMILY study. PLoS ONE.

[72] Ni M.Y., Li T.K., Pang H., Chan B.H., Kawachi I., Viswanath K., Schooling C.M., Leung G.M. (2016). Longitudinal patterns and predictors of depression trajectories related to the 2014 Occupy Central/Umbrella movement in Hong Kong. Am. J. Public Health.

[73] Çelebi E., Adam-Troian J., Mahfud Y. (2020). Positive links between exposure to police violence, PTSD, and depression symptoms among yellow vests protesters in France. J. Interpers. Violence.

[74] Lau J.T., Kim Y., Wu A.M., Wang Z., Huang B., Mo P.K. (2017). The Occupy Central (Umbrella) movement and mental health distress in the Hong Kong general public: Political movements and concerns as potential structural risk factors of population mental health. Soc. Psychiatry Psychiatr. Epidemiol..

[75] Dou D., Shek D.T.L. (2021). Concurrent and longitudinal relationships between positive youth development attributes and adolescent Internet addiction symptoms in Chinese mainland high school students. Int. J. Environ. Res. Public Health.

[76] Shek D.T.L., Dou D., Zhu X., Chai W. (2019). Positive youth development: Current perspectives. Adolesc. Health Med. Ther..

[77] Hameed N., Mehrotra S. (2017). Positive youth development programs for mental health promotion in Indian youth: An underutilized pathway. Int. J. Community. Med. Public Health.

[78] Hanton S., Mellalieu S.D., Hall R. (2004). Self-confidence and anxiety interpretation: A qualitative investigation. Psychol. Sport. Exerc..

[79] Frisch M.B. (1998). Quality of life therapy and assessment in health care. Clin. Psychol. N. Y..

[80] Paz C., Hermosa-Bosano C., Evans C. (2021). What happens when individuals answer questionnaires in two different languages. Front. Psychol..

[81] Solano P., Aguglia A., Caprino M., Conigliaro C., Giacomini G., Serafini G., Amore M. (2019). The personal experience of severe suicidal behaviour leads to negative attitudes towards self-and other’s suicidal thoughts and behaviours: A study of temperaments, coping strategies, and attitudes towards suicide among medical students. Psychiatry Res..

[82] Shek D.T.L. (2020). Protests in Hong Kong (2019–2020): A perspective based on quality of life and well-being. Appl. Res. Qual. Life.

[83] Shek D.T.L. (2021). COVID-19 and Quality of Life: Twelve Reflections. Appl. Res. Qual. Life.

[84] Lo S.M., Wong H.C., Lam C.Y., Shek D.T.L. (2019). An innovative multidisciplinary healthcare model in student mental health: Experience in Hong Kong. Appl. Res. Qual. Life.

